# Effectiveness of a Web- and Mobile-Guided Psychological Intervention for Depressive Symptoms in Turkey: Protocol for a Randomized Controlled Trial

**DOI:** 10.2196/13239

**Published:** 2019-04-05

**Authors:** Burçin Ünlü Ince, Didem Gökçay, Heleen Riper, Pim Cuijpers

**Affiliations:** 1 Department of Medical Informatics / Informatics Institute Middle East Technical University Ankara Turkey; 2 Ruhuna Iyi Bak Istanbul Turkey; 3 Section of Clinical Psychology Department of Clinical, Neuro and Developmental Psychology VU University Amsterdam Netherlands; 4 EMGO+ Institute for Health and Care Research VU Medical Centre Amsterdam Netherlands; 5 Department of Psychiatry VU Medical Centre/GGZ inGeest Amsterdam Netherlands; 6 Department of Clinical, Neuro and Developmental Psychology VU University Amsterdam Netherlands

**Keywords:** randomized controlled trial, depressive symptoms, mobile app, psychotherapy, telemedicine, depression

## Abstract

**Background:**

In Turkey, there are serious deficiencies in mental health care. Although depression is highly prevalent, only a small number of people seek professional help. Innovative solutions are needed to overcome this treatment gap. Web-based problem-solving therapy (PST) is an intervention proven to be effective in the treatment of depression, although little is known about its clinical effects in Turkey.

**Objective:**

This study aims to test the clinical effects of a Web and mobile app of an adapted PST for depressive symptoms among the general population in Turkey.

**Methods:**

Participants will be recruited through announcements in social media and the Middle East Technical University. Adults (18-55 years) with mild to moderate depressive symptoms (Beck Depression Inventory-II [BDI-II] score between 10-29) will be included in the study. Participants with a medium-to-high suicidal risk (according to the Mini-International Neuropsychiatric Interview) will be excluded. A 3-armed randomized controlled trial with a waiting control group will be utilized. A sample size of 444 participants will be randomized across 3 groups. The first experimental group will receive direct access to the Web version of the intervention; the second experimental group will receive direct access to the mobile app of the intervention as well as automated supportive short message service text messages based on PST. The control group consists of a wait-list and will gain access to the intervention 4 months after the baseline. The intervention is based on an existing PST for the Turkish population, *Her Şey Kontrol Altında* (HŞKA), consisting of 5 modules each with a duration of 1 week and is guided by a clinical psychologist. The primary outcome is change in depressive symptoms measured by the BDI-II. Secondary outcomes include symptoms of anxiety, stress, worry, self-efficacy, and quality of life. Furthermore, satisfaction with, usability and acceptability of the intervention are important features that will be evaluated. All outcomes will take place online through self-assessment at posttest (6-8 weeks after baseline) and at follow-up (4 months after baseline).

**Results:**

We will recruit a total of 444 participants with mild to moderate depressive symptoms from March 2018 to February 2019 or until the recruitment is complete. We expect the final trial results to be available by the end of May 2019. This trial is funded by the Scientific and Technological Research Council of Turkey (National Postdoctoral Research Fellowship Programme 2016/1).

**Conclusions:**

Results from this study will reveal more information about the clinical effects of HŞKA as well as its applicability in a Turkish setting through the Web and mobile platforms. On the basis of the results, a guided Web- and mobile-based PST intervention might become an appropriate alternative for treating mild to moderate depressive symptoms.

**Trial Registration:**

ClinicalTrials.gov NCT03754829; https://clinicaltrials.gov/ct2/show/NCT03754829 (Archived by WebCite at http://www.webcitation.org/74HugwLo7).

**International Registered Report Identifier (IRRID):**

DERR1-10.2196/13239

## Introduction

### Background

According to the Human Rights in Mental Health Initiative (RUSIHAK), mental health care in Turkey has serious deficiencies [1]. From 2011 to 2014, RUSIHAK monitored Turkey’s largest psychiatric hospitals and reported several problems. Among these are the undertreatment of mentally ill patients, a lack of psychologists, and inadequate psychosocial and rehabilitation options for patients and their relatives [1,2]. In Turkey, limited epidemiological studies are available; however, some studies revealed rates of depressive disorders ranging from 4.4% [3] up to 48.0% in the general population [4]. However, only 18% of these seek professional help during their lifetime [1]. Depression is one of the most common mental disorders worldwide [5].

The World Health Organization states that depression is the leading cause of disability globally [5]. It affects the quality of life considerably [6,7]; it is associated with impaired social relationships [8] and high economic costs [9,10]. Moreover, it is also associated with excess mortality rates [11].

Over the past decennia, several clinically effective treatments have been developed for depressive disorders, including antidepressant medication and psychotherapy such as cognitive behavioral therapy (CBT) [12-15]. However, not everyone who is in need of professional mental help receives adequate therapy in Turkey [1].

Innovative ways to overcome this treatment gap are electronic mental health (e-mental health) apps. E-mental health refers to the use of information and communication technologies for mental health, which are promising in several ways. The internet offers opportunities to target populations that are not sufficiently reached in other ways. Furthermore, self-management by the patient is encouraged, and the cost-effectiveness and delivery of treatments are increased [16]. As Turkey occupies fifth place among countries in Europe using the internet, with a penetration rate of 69.6% [17], and almost 84% of the Turkish population uses a smartphone [18], the internet could offer many benefits.

For approximately two decennia, Web-based treatments built on evidence-based face-to-face protocols, which are highly structured and guided, are being used [19]. These treatments have been found to be effective in the treatment of depression. The treatments include Web-based CBT [20] and problem-solving therapy (PST) [21]. The version of PST that is most examined as an internet-based intervention is based on self-examination therapy [22,23] and is aimed at teaching patients how to cope with solvable, unsolvable, and unimportant problems that cause depressive symptoms and determine the important things in life by a structured step-by-step method. By doing this, patients regain mastery of their problems in daily life, leading to a reduction in their depressive symptoms. However, only limited data on the effects of such interventions (either offered offline or online) in Turkish populations are available [24].

One randomized controlled trial (RCT) was conducted at the Vrije University in Amsterdam to test the clinical effects of a culturally adapted Web-based PST (originally developed by the VU University) *Her Şey Kontrol Altında* (HŞKA) for Turkish migrants with depression in the Netherlands [25]. A total of 287 people applied for the trial, but only 96 participants were included and randomized into 2 groups: 49 in the experimental group and 47 in the control group (wait-list). Results showed that there was no significant difference between the 2 groups concerning depression because of an underpowered sample. However, a high effect size was found at follow-up, suggesting possible effectiveness of the treatment in the longer term [25]. Furthermore, in a recent meta-analysis, it was found that PST is effective with small effect sizes across different populations and settings [21]. In addition, the internet can be also advantageous for several different purposes. To recruit participants, Facebook has been used in a Web-based intervention [25,26]. For example, Facebook has been used as part of the trial to reach Turkish migrants, which led to 3308 Friends on Facebook, of whom about 250 sent a direct message to the researcher [26], implying that the internet may be used to try to lower the stigma for seeking professional help for psychological problems. Electronic learning (e-learning) platforms can also be used for communicating about participants’ weekly exercises [24]. Videoconferencing can be applied as an efficient alternative to face-to-face therapy [27].

In 2014, the Vrije University in Amsterdam and Middle East Technical University (METU) in Ankara initiated a European scientific collaboration [28]. In a total of 11 European regions, computerized CBT and videoconferencing were evaluated and implemented in routine mental health care practices, with a minimum of 5230 patients with depression. The Web-based treatment HŞKA designed by the Vrije University, with the enhancement of a behavioral activation module, was used for the Turkey region. Initial results are encouraging regarding the reduction of depressive symptoms [24]. However, this intervention was provided through the student e-learning platform of the METU. This system was developed for coursework, in which users had to download and upload PDF or Word files to fulfill the exercise requirements of the Web-based therapy modules. For monitoring clients’ assessments and evaluations and to provide feedback, separate stand-alone software programs had to be used. Therefore, the overall suitability of an e-learning platform for Web-based PST was evaluated as low.

On another front, mobile health technologies may offer opportunities to boost the effects of psychological interventions [29]. For example, a recent study showed higher adherence in the experimental group who received CBT therapy with daily automated supportive messages on their smartphone compared with the control group who received CBT without these messages [30]. The addition of automated supportive short message service (SMS) text messages to Web-based interventions may be, therefore, potentially effective in monitoring patients and their adherence to treatment [30,31].

### Objectives

On the basis of our previous experience with HŞKA, we intend to start a new trial, because previous research has shown that computerized CBT is an effective way to reach and possibly to treat problems associated with mild to moderate depression [25,26]. In this trial, HŞKA as a stand-alone intervention will be used for 2 purposes. First, a compact Web and mobile app of the treatment will be developed and updated with pilot studies that evaluate the acceptance and usability of the intervention. Second, an RCT of the clinical effectiveness of the Web and mobile app will be conducted in the general population in Turkey.

## Methods

### Intervention Description

HŞKA was previously culturally adapted and tested for clinical effectiveness in Turkish migrants in the Netherlands [25,32]. The cultural adaptation consisted of language, the use of culture-specific cases and problems according to the worldview of the Turkish target group, and culture-specific examples of persons with similar problems. The intervention was translated from Dutch into Turkish, and it was adapted in terms of cultural sensitivity.

Cultural adaptation of psychotherapy has been defined as the modification of intervention protocols according to clients’ values, contexts, and worldviews [33]. Culture-specific adaptations in this intervention included several components: first, the participants’ native language; second, description of psychological problems in terms of idioms of distress (eg, using symptoms of depression instead of the term depression); third, explicit discussion of migration and culture by using culture-specific cases and problems that are recognizable for the target group concerned; and finally, inclusion of recognizable examples of persons with similar problems (eg, a young woman who migrated 2 years ago and cannot find her way in the Netherlands). For the purpose of this study, migration-related adaptations were removed, and the language was checked again by a native speaker.

HŞKA consists of 5 modules over 5 weeks, consisting of text, photos, videos, and exercises. Briefly, the length of each module is 1 page and the duration of videos 2 to 3 min. First, participants indicate what they think is important in their lives; they make a list of their problems and worries, and they categorize their problems into 3 groups: (1) unimportant problems, which are not related to what they think is important in their lives; (2) important but solvable problems, which are tackled with a systematic, problem-solving approach consisting of 6 steps (which consists of defining the problem, developing alternative solutions, choosing the best solution, making a plan for the solution, implementing the solution, and evaluation); and (3) important but unsolvable problems, such as having lost someone through death or having a chronic physical illness and making a plan for how to live with it. Each session includes about 1.5 A4 pages long text with explanations and examples and short videos about the theory; at the end of each session, exercises can be made. The core of the intervention is the 6-step problem-solving procedure, which teaches users this technique for several of their important but solvable problems. The idea is that by mastering this technique, people will regain mastery of their problems and ultimately their lives. Participants receive feedback on their homework assignments in brief weekly online messages from a clinical psychologist with a PhD degree in Clinical Psychology (first author). Those who have not sent their assignments will receive a reminder within the app once a week. After 3 reminders, participants who still have not sent anything will be considered as dropouts.

**Figure 1:**
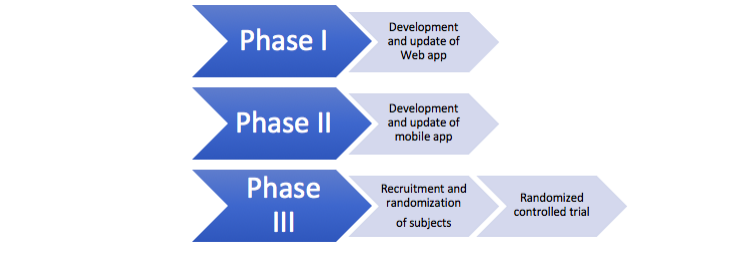
Flowchart of the study phases.

**Figure 2:**
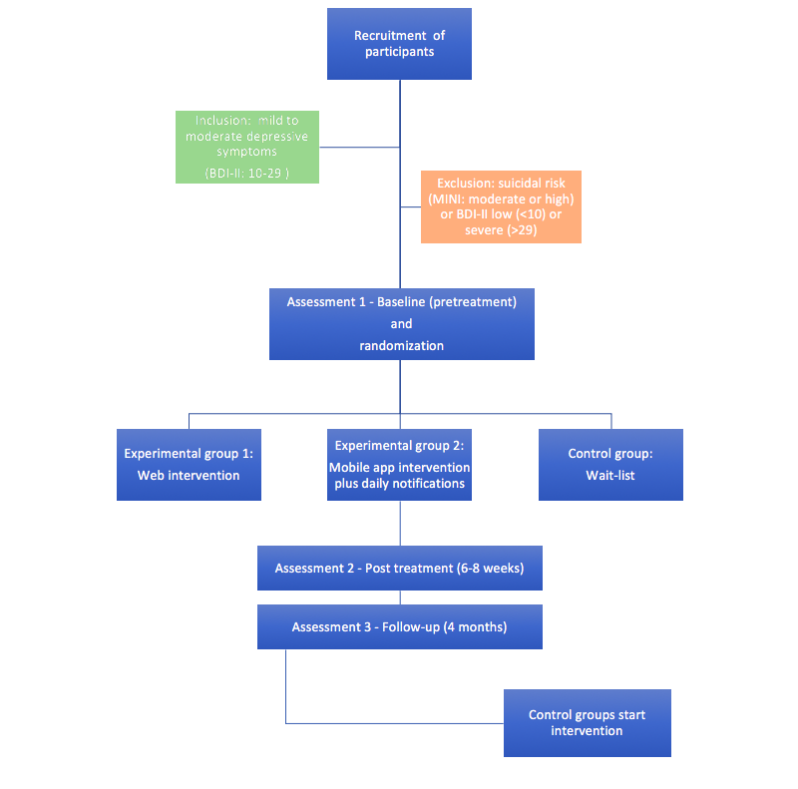
Flowchart of the randomized controlled trial. BDI-II: Beck Depression Inventory-II; MINI: Mini-International Neuropsychiatric Interview.

This project consists of 3 phases (Figure 1). In the first phase, the content of the existing intervention HŞKA for depressive symptoms will be used to develop a compact Web app. In the second phase, the content of the Web-based intervention will be used to develop a mobile app. In both of these phases, pilot studies will be conducted to optimize the software apps to find and resolve software errors. In this last phase, the final version of the Web and mobile apps will be used to perform an RCT with 3 arms. Screenshots of the interventions are provided in Multimedia Appendices 1-3 [34]. The first experimental group will receive direct access to the Web version of the intervention; the second experimental group will receive direct access to the mobile app of the intervention and will also receive daily automated supportive SMS text messages based on PST once a day for 2 months. The content in the daily notifications is derived from the content of the modules, for example, “Once a week, take a birds-eye view of your life to evaluate it.” The rationale behind using notifications in the mobile app is to avoid dropouts as indicated [30,31]. Adding notifications for the Web version does not make it equal to the mobile version because the users who use the Web version only see the notifications if they decide to log in, whereas the users of the mobile version see the notifications when a flag appears on their main screen. The control group consists of a wait-list and will receive the treatment after the study has been finished. A flow chart of the RCT is presented in Figure 2. Ethical approval for the RCT study has been obtained from the Human Subjects Ethics Committee of METU (No. 28620816/21).

### Inclusion and Exclusion Criteria

Turkish participants living in Turkey are eligible if they are aged between 18 and 55 years, have internet access, have both a personal computer (PC) and a smartphone or tablet, and have mild to moderate depressive symptoms (Beck Depression Inventory-II [BDI-II] score between 10 and 29) [35]. Participants will be excluded if they have a BDI-II score above 29 or a medium-to-high suicidal risk (according to the Mini-International Neuropsychiatric Interview [MINI]), and they are advised to contact a psychiatrist or clinical psychologist by sending them an email [36,37].

### Recruitment

Recruitment will take place in the general population in Turkey until the end of the project or earlier if the sample size will be achieved. A total of 2 psychologists will be involved in the recruitment, assessments, and therapy delivery: the clinical psychologist will deliver the therapy messages, follow up with the administration of the assessments, message management for admission to the study, and convince dropouts to continue, whereas the other psychologist (Bachelor’s degree) will actively recruit participants for the study. Announcements will be placed online at health-related social media sites and offline at several meeting points at the university. These announcements will contain a link to the research website with detailed information about the study. Respondents who are interested can apply by sending an email to the clinical psychologist. A link to the screening and an informed consent form will be emailed to the respondent. The screening includes the BDI-II and MINI questionnaires; access to a PC and smartphone or tablet; and demographic information of the respondents, such as name, surname, email address, age, and gender. Applicant admission is done by checking the BDI-II and MINI scores of applicants manually.

### Measures

Assessments will take place before randomization (baseline), directly after completing the treatment (2 months after baseline), and finally, 4 months after baseline as a follow-up assessment. All outcomes are self-assessed through online questionnaires and can take up to 20 to 30 min to complete. Participants will receive 15 TL (Turkish Lira, the local currency) in total only if completing all 3 assessments. An overview of the measures used per assessment is given in Table 1.

#### Sociodemographic and Additional Information

At baseline, sociodemographic information about the participant will be collected by asking questions about the following: sex, age, educational level, employment, long-term relationship or partner status, living situation, chronic physical illness, chronic psychological illness, and psychological or psychiatric treatment status.

#### Primary Outcome: Depressive Symptoms

To measure depressive symptoms as the primary outcome, the Turkish version of the self-report BDI-II will be used at baseline, posttreatment, and follow-up [38]. It has 21 items in total describing depressive symptoms. Each item is scored on a 4-point scale, with a range of 0 (not at all) to 3 (severe). The total score ranges from 0 to 63. The BDI-II has good psychometric properties for online administration [39]. The Turkish version has been proven to have good reliability and validity [38,40]. The cut-off scores are 0 to 9 for minimal, 10 to 29 for moderate, and 30 to 63 for severe depression.

#### Secondary Outcomes

##### Anxiety Symptoms

Anxiety will be measured with the State-Trait Anxiety Inventory (STAI), a measure of trait and state anxiety by self-report [41]. The STAI has 40 items in total, of which 20 items are allocated in the state scale (S-Anxiety) and 20 items in the trait scale (T-Anxiety). The S-Anxiety scale is intended to assess the intensity of current feelings of anxiety, whereas the T-Anxiety scale assesses feelings of anxiety in general. All items are rated on a 4-point scale (from almost never to almost always). A higher score indicates greater anxiety levels. The STAI is available in Turkish and has been shown to be a valid and reliable measure [42].

**Table 1:** Overview of instruments per assessment.

Instruments	Number of items	Baseline	Posttreatment	Follow-up
Sociodemographic and additional information	20	✓^a^	—^b^	—
Depressive symptoms (Beck Depression Inventory-II)	21	✓	✓	✓
Anxiety (State-Trait Anxiety Inventory)	40	✓	✓	✓
Worry (Penn State Worry Questionnaire)	16	✓	✓	✓
Stress (Perceived Stress Scale)	10	✓	✓	✓
General Self-Efficacy	10	✓	✓	✓
Quality of life (EuroQol-5D-5L)	6	✓	✓	✓
Satisfaction and log data	8	—	✓	—
Usability and acceptability	40	—	✓	—

^a^These instruments will be assessed during the trial.

^b^These instruments will only be assessed once during the trial.

##### Worry

The Penn State Worry Questionnaire (PSWQ) will be used to measure pathological worry [43]. The PSWQ has 16 items in total. Each item can be scored on a 5-point scale from 1 (not at all typical of me) to 5 (very typical of me), with a total score varying from 16 to 80. The PSWQ has good psychometric properties to use in an online format [44]. The PSWQ has been translated into Turkish and shown to be valid and reliable in a Turkish sample [45].

##### Stress

The Perceived Stress Scale (PSS) will be used to measure the perception of stress [46]. It consists of 14 items originally, but it can also be assessed in a short form of 10, for which the total score range is 0 to 40 [47]. Each item is rated on a 5-point scale, ranging from 0 (never) to 4 (very often). The Turkish version of the PSS has been shown to be valid and reliable [48]. For this study, the short form consisting of 10 items will be used.

##### Self-Efficacy

The General Self-Efficacy (GSE) scale is a measure to assess self-efficacy beliefs [49]. The GSE scale contains 10 items, which are rated on a 4-point scale from 1 (not at all true) to 5 (exactly true). The total score varies from 10 to 50. The Turkish version will be used, which has been shown to have good reliability and validity [50].

##### Quality of Life

To measure quality of life, the EuroQol-5D-5L (EQ-5D-5L) will be used [51]. It consists of 5 items each measuring different dimensions of health status (mobility, self-care, usual activities, pain or discomfort, and anxiety/depression). The items are rated on a 5-point scale from level 1 to level 5 (no problems, slight problem, moderate problem, severe problem, and extreme problem). All the answers to each item are combined, resulting in 3125 possible health states, ranging from 11,111 (full health) to 55,555 (worst health). Furthermore, there is an EQ-visual analog scale measuring a global rating of self-perceived health. This is scored by 0 (the worst health you can imagine) to 100 (the best health you can imagine). The EQ-5D-5L is valid and reliable for use in an online format [52]. The Turkish version has good validity and reliability properties [53].

##### Satisfaction and Log Data

After completion of the intervention, participants will be asked to define their satisfaction with each module in the intervention (ie, “Was this lesson useful to you?”). The answers can be rated on a 5-point Likert scale from 1 (not at all) to 5 (very useful). Finally, log data will be collected by using the following parameters: type of device, last activity, first session, app version, and usage duration, which will be monitored until the follow-up assessment.

##### Usability and Acceptability

To test the final version of the Web and mobile app of the intervention, the usability and acceptability will be measured. A questionnaire developed by Çetin Kaya [54] about acceptance of the technology will be used. It consists of 3 parts: first, the general use of electronic services (e-services) and second, the acceptance of the e-service concerned, which consists of 40 items (of which 2 have been removed from this study because of irrelevance). The questionnaire has 13 subscales: perceived application mobility, perceived device mobility, expected benefit, informational influence, intention to use e-service, perceived behavioral control, perceived enjoyment, value to personalization, perceived usability, trust in the e-service, perceived ubiquity, value expressiveness, and value to incentive. This questionnaire has good psychometric properties in terms of validity and reliability [54].

### Sample Size

In this RCT, 2 experimental groups (receiving the intervention by either the internet or a mobile app) will be compared with a waiting list control group. The sample size is calculated based on an expected difference of Cohen *d*=0.45 (moderate) between 1 of the experimental groups and the control group as in previous studies [22,25]. “ *F* tests-analysis of variance repeated measures” will be applied in G-Power Statistical Power Analysis program version 3.1 and used for sample size calculation [55]. To obtain a power of .80 for a 1-tailed test and an alpha of .05, a total of 234 participants for 3 groups is needed. However, high attrition rates at posttest (25.0%) and at follow-up (30.0%) are expected based on previous studies [24,25]. Therefore, the sample size will be increased to 444 participants in total (allowing an attrition of 25.0%*30.0%=52.5%). This means that 148 participants are required at baseline for each group.

### Randomization

Participants will be randomly assigned to 1 of the 2 experimental groups or to the control group after the first assessment (at baseline) based on 1:1:1 randomization. The allocation schedule will be generated using an online randomization tool [56], which will be performed and communicated to the patient by an independent researcher. Block randomization will be used with blocks of 9 allocations each. Furthermore, 2 strata will also be used based on age: 1 stratum for 18 to 24 years and another for 25 to 55 years. Participants will be informed about the randomization outcome by email after completing the baseline assessment. Participants in the experimental groups receive a Web link to subscribe free to the intervention with a special code. Afterwards, the participants are required to create an account on the platform with their personal code and self-chosen password. The mobile app will be free to download in the App Store and Google Play Store; however, the intervention will not be open to the general public. Blinding the participants in this RCT will not be possible; however, the statistician is blinded to group assignment.

### Statistical Analyses

The study will be reported in accordance with the consolidated standards of reporting trials guidelines [57]. The data obtained from the study will be evaluated and data tables created using the Statistical Package for Social Sciences version 25.0 statistical software program [58]. The significance level will be accepted as 95% CI (*P*<.05) in all statistical analyses. Quantitative (categorical) variables will be expressed as mean, SD, and upper and lower values in this study, whereas qualitative (continuous) variables will be expressed as numbers (n) and percentage (%). The Kolmogorov-Smirnov Test will be used to examine whether the quantitative data were distributed normally. To measure the efficacy of the treatment in this study, general linear model repeated measures analysis will be used to investigate the change on the primary outcome (BDI-II) scores in dependent variables for the total sample based on the intention-to-treat principle and for analyzing missing data [59]. McNemar tests will be used to measure categorical change as a version of the Chi-square test of independence in repeated measures of dependent groups. Furthermore, the Fisher exact Chi-square tests will be used if the number of crosstabs data is insufficient and the hypothesis is not achieved. Attrition will be defined as not completing any or 1 of the posttreatment measures. The notification effect will be analyzed by comparing the dropout rates of the Web and mobile versions. Finally, the correlation coefficient and statistical significance between the quantitative variables will be calculated by 2-way Pearson correlation analysis.

### Clinically Significant Change

Analyses of clinically significant change on the primary outcome (BDI-II) will be conducted according to the Jacobson and Truax formula [60]. This method evaluates 2 criteria for each participant. The first is whether each participant’s BDI-II score has improved such that it is unlikely to be because of chance (Reliable Change Index [RCI]). The RCI is a function of a participant’s pretest and posttest scores, the SD of the population before treatment, and the test-retest reliability of the measure [55]. A participant is considered to have experienced reliable change if his or her RCI is greater than 1.96 [61]. The second criterion evaluated for participants shown to have reliable change is whether their posttreatment symptom level places them at a score of 10 or lower on the BI-II. A clinically significant change was determined if the participant had recovered and shown reliable improvement over time.

### Per-Protocol Analysis

Secondary per-protocol analyses will be performed for participants who completed all the measurements and all 5 modules of the intervention (if randomized to the experimental conditions).

## Results

A total of 444 participants with mild to moderate depressive symptoms will be recruited from March 2018 to February 2019 or until the recruitment is complete. We expect the final trial results to be available by the end of May 2019. This trial is funded by the Scientific and Technological Research Council of Turkey under its National Postdoctoral Research Fellowship Programme 2016/1.

## Discussion

### Strengths

In an earlier report, RUSIHAK raised alarm concerning mental health care in Turkey [1]. A new mind-set and alternative solutions for this challenging problem are needed. E-mental health could be an inventive solution to reach, treat, and monitor people with psychological problems. The flexibility in time and place and the high levels of anonymity and accessibility could make a positive difference to mental health care.

To our knowledge, this project will be the first trial studying the effects of guided PST in a Web-based and mobile app for depressive symptoms in the general population in Turkey. In addition, the effects of receiving notifications based on PST techniques in the app will be evaluated. Although notifications seem to be a promising solution to increase adherence, the impact of it has earlier been evaluated as disruptive [62]. As Turkey has some major shortcomings in providing mental health care, HŞKA can bridge this gap in several ways, such as providing alternative and evidence-based care besides pharmacotherapy and relatively easily accessible health care for a wider population in need of help. Online guided self-help can be an innovative way to reach and treat populations with limited access to mental health care. The first and second phases of the study will shed light on the general stance of the Turkish population toward mental health and the usability and acceptability of the apps.

### Limitations

Although online guided self-help for depressive symptoms is promising, there are some points to consider. First, in a previous study among Turkish migrants with depressive symptoms, the sample size was not large enough to detect a small to moderate effect [25]. Recruitment was difficult, and the suicidal risk was moderate to high in the same study. It is expected that the same difficulties will be encountered during this trial. Second, attrition is generally high in online intervention trials and can be more than 50% [63,64]. In the study of Ünlü Ince et al [25], attrition was even higher, which led to missing data. It is expected that attrition in this study will be comparable with that of online intervention studies; however, the daily automated notifications in the mobile experimental group may counteract such high attrition rates. Finally, this study will only evaluate the severity of depressive symptoms, which means it will not be possible to say anything about recovery from a depressive disorder.

### Conclusions

In the event that promising results are obtained from this study, usage of Web and mobile apps in a Turkish setting will be validated. Thereby, guided Web and mobile-based PST intervention might become an appropriate alternative to psychiatric care for mild to moderate depressive symptoms. In the future, the feasibility, economics, and management of online apps for depression will be studied.

## Appendix

Multimedia Appendix 1 Screenshot of the main page of the Web version.

Multimedia Appendix 2 Screenshot of module 1 of the mobile version.

Multimedia Appendix 3 Screenshot of the exercise mobile version.

## Abbreviations

BDI-II: Beck Depression Inventory-II
CBT: cognitive behavioral therapy
EQ-5D-5L: EuroQol-5D-5L
GSE: General Self-Efficacy
HŞKA: Her Şey Kontrol Altında
METU: Middle East Technical University
PC: personal computer
PSS: Perceived Stress Scale
PST: problem-solving therapy
PSWQ: Penn State Worry Questionnaire
RCI: Reliable Change Index
RCT: randomized controlled trial
RUSIHAK: Human Rights in Mental Health Initiative
SMS: short message service
STAI: State-Trait Anxiety Inventory
