# Supplementary figures and images for: Effectiveness of a Web- and Mobile-Guided Psychological Intervention for Depressive Symptoms in Turkey: Protocol for a Randomized Controlled Trial
Source: JMIR Res Protoc. 2019 Apr 5;8(4):e13239. doi: 10.2196/13239 (PMC6473215; doi:10.2196/13239)

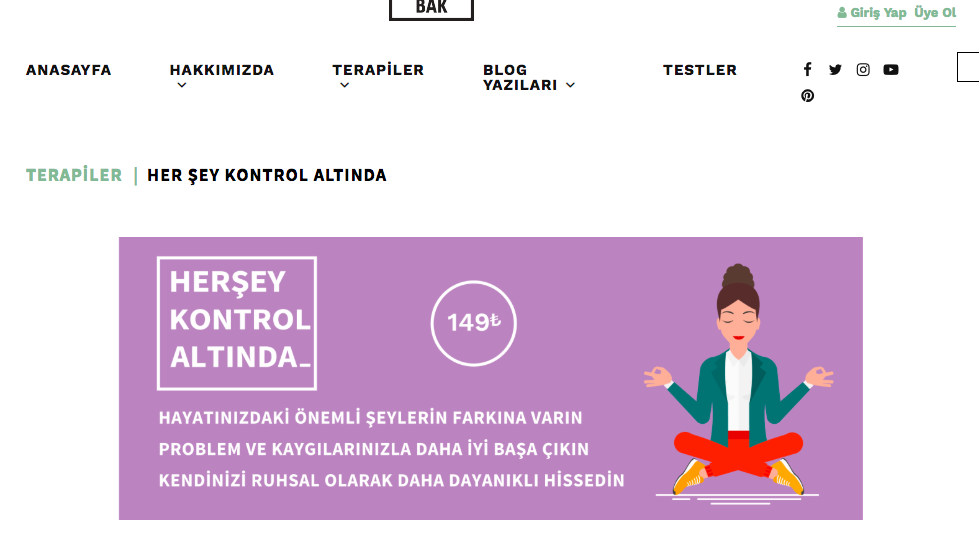

Supplement: Multimedia Appendix 1 [file resprot_v8i4e13239_app1.png]

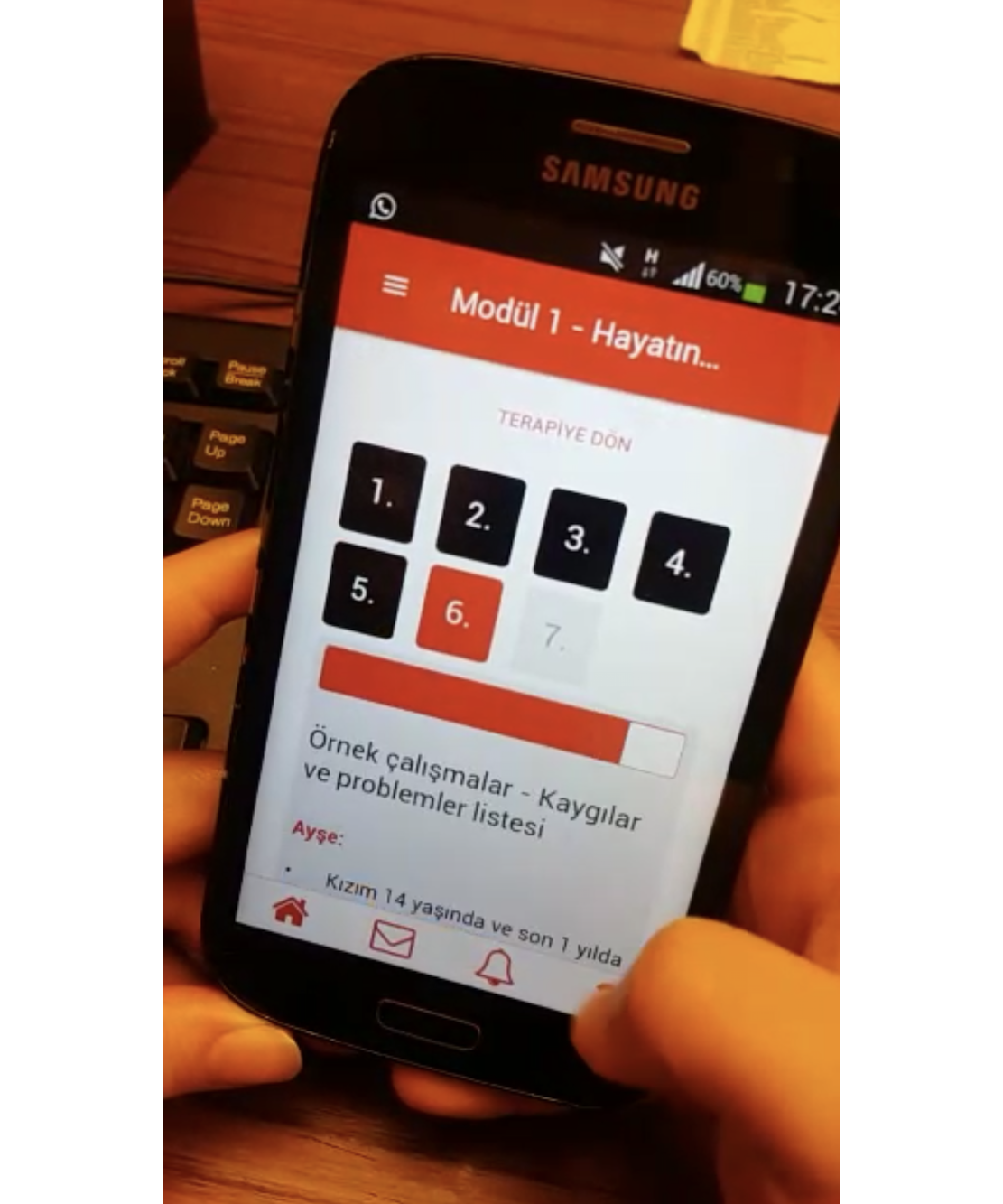

Supplement: Multimedia Appendix 2 [file resprot_v8i4e13239_app2.PNG]

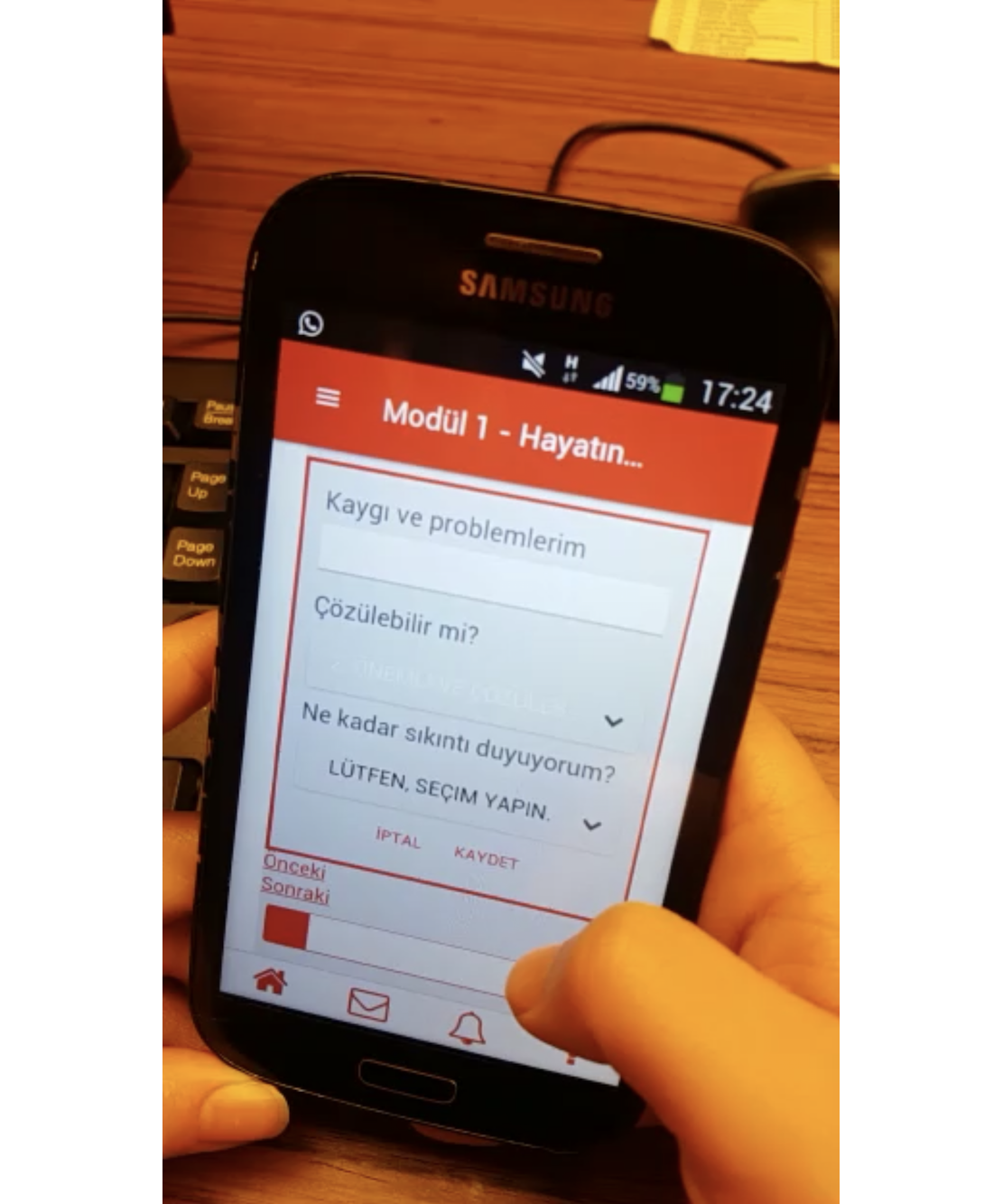

Supplement: Multimedia Appendix 3 [file resprot_v8i4e13239_app3.PNG]
